# Refractory postoperative chronic rhinosinusitis and otitis media as the initial clue to hypogammaglobulinemia and *Pneumocystis jirovecii* pneumonia: A case report

**DOI:** 10.1016/j.rmcr.2026.102536

**Published:** 2026-10-27

**Authors:** Ryota Higuchi, Yusuke Miyamoto, Kenichi Nakao, Shunsuke Tanaka, Yoshihiro Eriguchi, Takashi Nakagawa

**Affiliations:** a Department of Otorhinolaryngology–Head and Neck Surgery, Graduate School of Medical Sciences, Kyushu University, 3-1-1, Maidashi, Higashi-ku, 815-8582, Fukuoka, Japan; b Department of Clinical Immunology and Rheumatology/Infectious Disease, Kyushu University Hospital, 3-1-1, Maidashi, Higashi-ku, 815-8582, Fukuoka, Japan

**Keywords:** Chronic rhinosinusitis, Hypogammaglobulinemia, *Pneumocystis jirovecii* pneumonia, *Pseudomonas aeruginosa*, Case report

## Abstract

Chronic rhinosinusitis is a common disease entity in otolaryngology; however, refractory chronic rhinosinusitis accompanied by recurrent otitis media or persistent upper-airway infection may suggest an underlying systemic immune disorder. We present a 69-year-old man with persistent purulent rhinorrhea and postnasal drip despite two prior endoscopic sinus surgeries, who later developed left-sided otitis media with tympanic membrane perforation. He had a history of diffuse large B-cell lymphoma that was treated with surgery and rituximab-containing chemotherapy. Cultures of nasal secretions and otorrhea yielded *Pseudomonas aeruginosa*, while immunological evaluation revealed hypogammaglobulinemia and elevated serum β-D-glucan. Following admission, the sinonasal and otologic findings improved with intensive local care, systemic antimicrobial therapy, and tympanostomy tube placement. During hospitalization, however, hypoxemia requiring supplemental oxygen was recognized, and chest computed tomography revealed bilateral upper lobe-predominant ground-glass opacities. The combination of hypoxemia, bilateral ground-glass opacities, elevated beta-D-glucan, and a positive sputum *Pneumocystis jirovecii* polymerase chain reaction result supported the clinical diagnosis of *Pneumocystis jirovecii* pneumonia. Trimethoprim-sulfamethoxazole and systemic corticosteroids were administered, together with a single dose of intravenous immunoglobulin for hypogammaglobulinemia. Respiratory and otolaryngological symptoms subsequently improved. This case suggests that refractory sinonasal and otologic infections may prompt earlier evaluation for underlying immune dysfunction and opportunistic infections.

## Highlights

• Refractory postoperative CRS with otitis media prompted immune evaluation.
• CT findings, β-D-glucan, and sputum PCR supported a clinical PJP diagnosis.
• ENT infections may precede recognition of opportunistic pulmonary infection.

## 1 Introduction

Chronic rhinosinusitis (CRS) is a common inflammatory disorder encountered in otolaryngology practice. Although most patients respond to appropriate medical therapy, and when indicated, endoscopic sinus surgery, a subset experiences persistent or recurrent symptoms despite repeated interventions [1]. In such situations, especially when purulent discharge persists after surgery or when otologic involvement is present, the clinical course may not be adequately explained by local disease alone.

Immune dysfunction is increasingly recognized as an important predisposing factor for refractory CRS [2]. Humoral immunodeficiency predisposes patients to recurrent infections of the upper and lower respiratory tract, and delayed recognition may lead to significant morbidity, including chronic bronchopulmonary disease and opportunistic infection [3]. Patients with a history of hematological malignancy or rituximab-containing chemotherapy may develop prolonged secondary hypogammaglobulinemia associated with a clinically significant susceptibility to infection [4], [5].

We report a case of postoperative refractory CRS in which persistent sinonasal and otological infections led to the diagnosis of hypogammaglobulinemia with CD4/CD8 lymphopenia of undetermined etiology, and a subsequent clinical diagnosis of *Pneumocystis jirovecii* pneumonia (PJP) supported by pulmonary and microbiological findings. This case underscores the important role of otolaryngologists in considering an underlying immune disorder in treatment-resistant upper airway infections.

## 2 Case report

A 69-year-old man was referred to our department with persistent purulent rhinorrhea, postnasal drip, productive cough, and left-sided otorrhea. His medical history was notable for diffuse large B-cell lymphoma involving the ileocecal region. Ten years prior to the current presentation, he underwent surgical resection followed by three cycles of rituximab, cyclophosphamide, doxorubicin, vincristine, and prednisolone (R-CHOP), after which he remained in complete remission. Other comorbidities included dilated cardiomyopathy, chronic heart failure, prior septoplasty, and transient ischemic attacks.

One year before presentation, he developed persistent rhinosinusitis and underwent two endoscopic sinus surgeries at another hospital. Despite these procedures, purulent nasal discharge and postnasal drip continued. One month before referral, the patient developed left otitis media with otorrhea. Myringotomy and antibiotic therapy at a local clinic did not provide adequate improvement, and he was referred to our hospital for further evaluation and management.

At the initial visit, nasal endoscopy revealed a large amount of purulent discharge in both the middle meatuses (Fig. 1A). Otoscopic examination revealed a small perforation in the left tympanic membrane with active otorrhea (Fig. 1B). Paranasal sinus computed tomography (CT) revealed postoperative changes and mild mucosal thickening involving the bilateral maxillary, ethmoid, frontal, and sphenoid sinuses (Fig. 1C). Poor aeration with opacification was also noted in the middle ear and mastoid cavity (Fig. 1D).

**Fig. 1:**
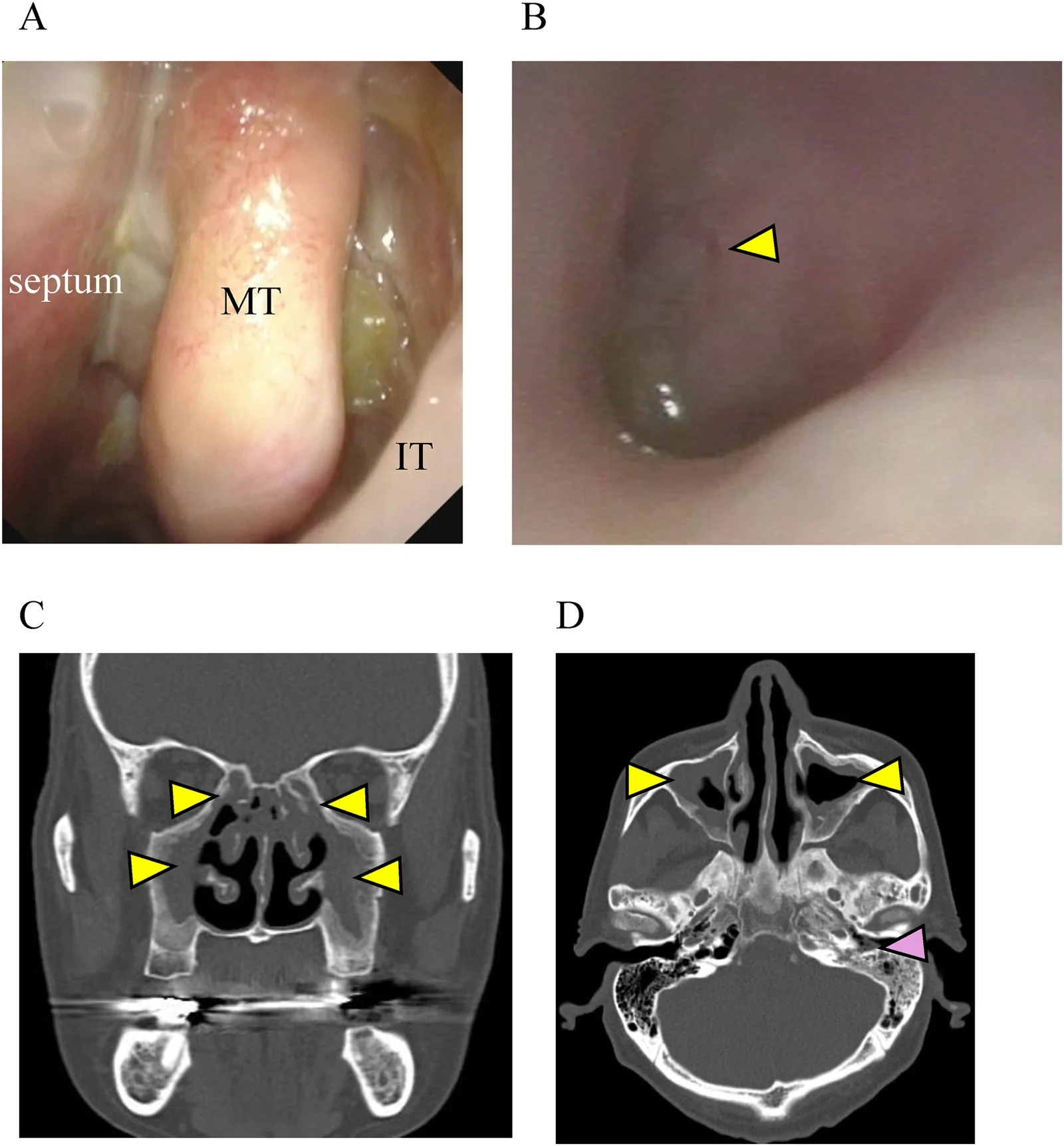
Initial endoscopic, otoscopic, and computed tomography findings. (A) Nasal endoscopy demonstrated abundant purulent discharge in both middle meatuses. (B) Otoscopic examination revealed a small perforation of the left tympanic membrane with otorrhea (yellow arrowhead). (C, D) Computed tomography demonstrated findings consistent with sinusitis (yellow arrowhead) and left otitis media (violet arrowhead). Abbreviations: MT, middle turbinate; IT, inferior turbinate.

Because outpatient management, including oral levofloxacin, was ineffective and underlying immune dysfunction was suspected, the patient was admitted to the Department of Otolaryngology. Intensive local treatment, including nasal irrigation and ear toilets, was initiated together with systemic antimicrobial therapy using cefepime, and tympanostomy tube placement. Cefepime was administered at a dose of 1 g three times daily. Following admission, the sinonasal and otological findings gradually improved. However, dyspnea and hypoxemia were evident after hospitalization. His oxygen saturation on room air decreased to a low 80% range, and supplemental oxygen at 3–5 L/min was required. At presentation, SpO2 was 90-95% while receiving supplemental oxygen at 3 L/min. Arterial blood gas analysis performed while receiving oxygen at 4 L/min showed a pH of 7.437, PaO2 of 149.0 mmHg, and PaCO2 of 36.8 mmHg. Bacterial cultures from both nasal discharge and otorrhea yielded *Pseudomonas aeruginosa*. Immunological evaluation revealed hypogammaglobulinemia with a serum IgG level of 590 mg/dL, IgA level of 78 mg/dL, and IgM level of 14 mg/dL (Table 1). Laboratory testing also showed an elevated KL-6 level of 1142 U/mL and a serum β-D-glucan level of 92.09 pg/mL (Table 1). The serum β-D-glucan level subsequently increased to 205.4 pg/mL and decreased during treatment (89.82 and 85.78 pg/mL on subsequent measurements). Chest CT demonstrated bilateral upper lobe-predominant centrilobular ground-glass opacities with scattered small nodules (Fig. 2). The combination of hypoxemia, bilateral ground-glass opacities, elevated β-D-glucan, and a positive sputum *Pneumocystis jirovecii* polymerase chain reaction (PCR) result supported a clinical diagnosis of PJP. Quantitative real-time PCR of spontaneously expectorated sputum detected *P. jirovecii* DNA at 2 × 10^5^ copies/μgDNA (reference value: <40 copies/μgDNA; BML, Inc., Tokyo, Japan).

**Table 1:** Laboratory findings at initial evaluation. Reference ranges are institutional where available. CD4/CD8 values were obtained after corticosteroid initiation. Arterial blood gas (ABG) analysis was performed while the patient was receiving supplemental oxygen at 4 L/min. The reference range for PaO_2_ on room air was not applicable.

Item	Result	Reference range	Unit
Nasal discharge culture/otorrhea culture	*Pseudomonas aeruginosa*	-	-
WBC	5000	3300–8600	/μl
Lymphocyte (absolute count)	690		/μl
LDH	305	124-222	U/L
Creatinine	0.8	0.65-1.07	Mg/dL
Albumin	3.7	4.1-5.1	g/dL
β-D-glucan	92.09	-	pg/mL
IgG	590	861-1747	mg/dL
IgM	14	33-183	mg/dL
IgA	78	93-183	mg/dL
KL-6	1142	500	U/mL
CD4-positive T-cell count	204	-	/μL
CD8-positive T-cell count	68	-	/μL
HIV antibody	Negative	-	-
T-SPOT	Negative	-	-
*Pneumocystis jirovecii* DNA	2 × 10^5^	40	copies/μgDNA
ABG pH (O2 4 L/min)	7.437	7.35–7.45	
ABG PaO2 (O2 4 L/min)	149	-	mmHg
ABG PaCO2 (O2 4 L/min)	36.8	35-45	mmHg
ABG HCO_3_^−^ (O2 4 L/min)	24.8	22-26	mmol/L

**Fig. 2:**
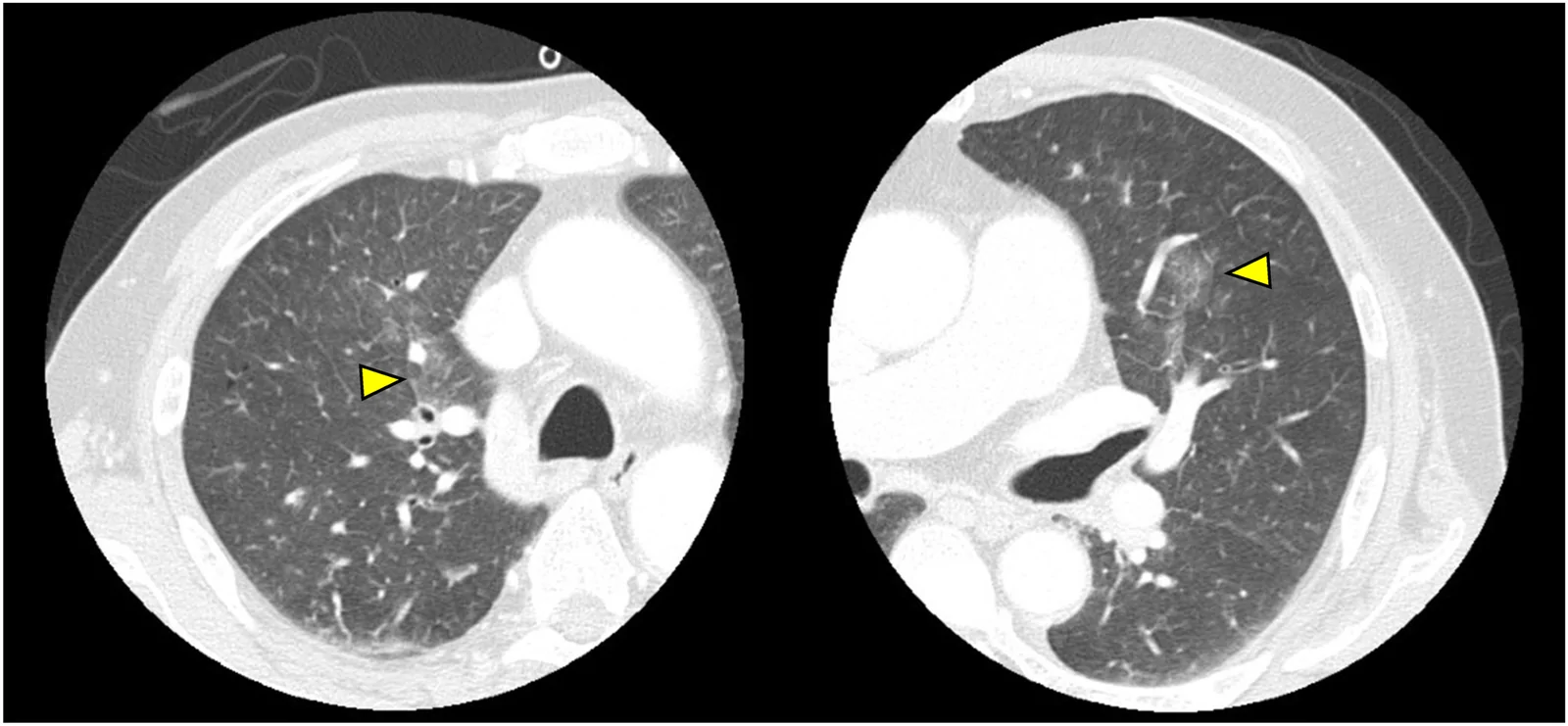
Chest computed tomography findings. Chest computed tomography demonstrated bilateral upper-lobe-predominant centrilobular ground-glass opacities and small nodular opacities (yellow arrowhead).

Lymphocyte subset analysis, performed after initiation of systemic corticosteroids, showed CD4-positive T cells of 204/μL and CD8-positive T cells of 68/μL. HIV-1/2 antibody confirmatory testing (BML, Inc., Tokyo, Japan) was negative. Taken together, the clinical findings raised concern for underlying immune dysfunction. The patient was characterized as having hypogammaglobulinemia with CD4/CD8 lymphopenia of undetermined etiology.

The patient was transferred to the Department of Infectious Diseases and received trimethoprim-sulfamethoxazole (TMP-SMX) at 12 mg/kg/day for 21 days. Owing to the emergence of adverse events such as renal dysfunction, the dosage was stepped down incrementally, eventually reaching 8 mg/kg/day. Prednisolone was started at 80 mg/day after specialist consultation because of clinically significant hypoxemic respiratory impairment and was gradually tapered (Fig. 3). His respiratory condition gradually improved, oxygen therapy was discontinued, and the purulent rhinorrhea, postnasal drip, and otorrhea resolved completely. The patient received a single dose of intravenous immunoglobulin (IVIG; Privigen 10%, 20 g/200 mL) during hospitalization for hypogammaglobulinemia. Serum IgG decreased from 590 mg/dL at initial evaluation to 451 mg/dL before IVIG. The patient was discharged in stable condition. Six weeks after administration, the serum IgG level increased to 645 mg/dL, although it remained slightly below the reference range. Because the patient resided far from our hospital, follow-up care was transferred to a local medical institution. (Table 2).

**Fig. 3:**
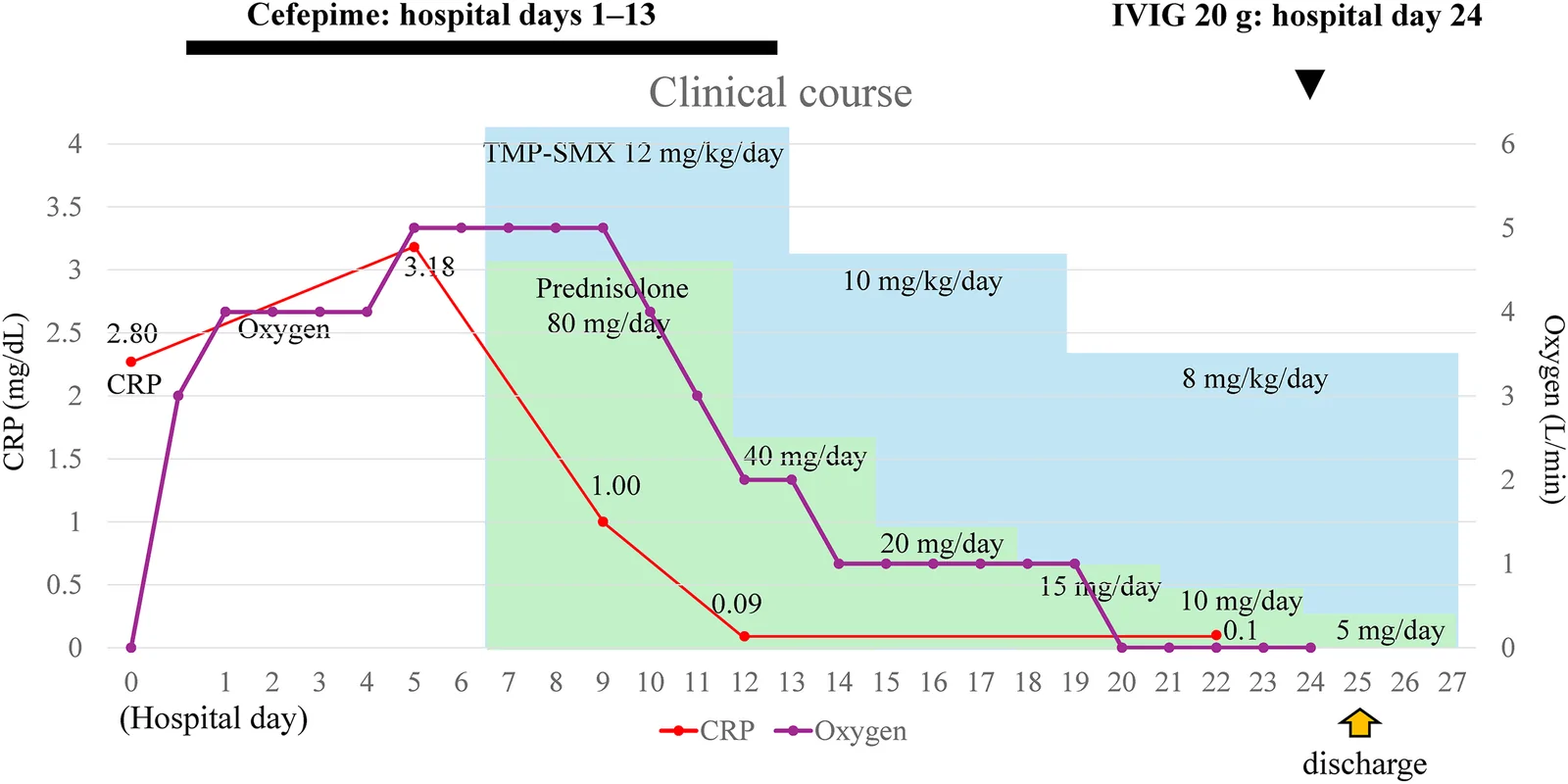
Clinical course. Timeline of C-reactive protein levels, supplemental oxygen requirement, cefepime (hospital days 1–13), TMP-SMX (21-day treatment course), systemic corticosteroids, and a single IVIG dose (20 g on hospital day 24). All TMP-SMX doses in mg/kg/day refer to the trimethoprim component. Hospital days are counted from admission to the Department of Otolaryngology.

**Table 2:** Clinical timeline. Timeline from previous lymphoma treatment through r Refractory rhinosinusitis, PJP evaluation and therapy, IVIG, discharge, and available six-week follow-up. Subsequent 3–6-month immunologic outcomes were unavailable. Dates are represented by relative intervals or hospital days.

Time	Clinical findings and assessment	Interventions and outcomes
Approx. 10 years before referral	Ileocecal diffuse large B-cell lymphoma	Surgical resection and three cycles of R-CHOP Complete remission
Approx. 1 year before referral	Persistent chronic rhinosinusitis	Two endoscopic sinus surgeries Purulent rhinorrhea and postnasal drip persisted
Approx. 1 month before referral	Left otitis media with otorrhea	Myringotomy and antibiotics Inadequate improvement
Initial visit	Purulent rhinorrhea and left otorrhea *P. aeruginosa* from both sites. IgG 590 mg/dL	Outpatient antibiotics, including levofloxacin Persistent symptoms prompted admission
Hospital day 1	Admission for refractory sinonasal and otologic infection	CFPM, nasal irrigation and ear cleaning Tympanostomy tube placement
Early admission	Hypoxemia and bilateral ground-glass opacities β-D-glucan 205.4 pg/mL Sputum *P. jirovecii* PCR positive (2 × 10^5^ copies/μg)	Supplemental oxygen Overall findings supported a clinical diagnosis of PJP
Hospital day 8	PJP treatment initiated	TMP-SMX for 21 days (initial TMP 12 mg/kg/day) Prednisolone 80 mg/day, followed by tapering
Hospital day 16	Respiratory condition improved	Supplemental oxygen discontinued
Hospital day 20	IgG 451 mg/dL β-D-glucan decreased to 85.78 pg/mL	
Hospital day 24	Persistent hypogammaglobulinemia	Single dose of IVIG (20 g) No infusion-related adverse effects
Hospital day 25	Respiratory improvement Sinonasal and otologic symptoms resolved	Discharged in stable condition TMP-SMX and steroid taper continued at home
Hospital day 28 and thereafter	Completion of the 21-day PJP treatment course Completion of the corticosteroid	Subsequent prophylactic TMP-SMX
6 weeks after discharge	IgG 645 mg/dL Increased, but remained below the normal range Etiology of immune dysfunction remained undetermined	Subsequent follow-up at another hospital No further clinical or immunologic data available

Hospital day 1 denotes admission to the Department of Otolaryngology. Pre-referral intervals are approximate.

The sputum specimen for *P. jirovecii* PCR was collected on hospital day 8.

PJP, *Pneumocystis jirovecii* pneumonia. PCR, polymerase chain reaction. IgG, immunoglobulin G.

TMP-SMX, trimethoprim-sulfamethoxazole. TMP, IVIG, intravenous immunoglobulin.

R-CHOP, rituximab, cyclophosphamide, doxorubicin, vincristine and prednisolone.

## 3 Discussion

This case illustrates how refractory sinonasal and otologic infections in a patient with a history of lymphoma treatment prompted evaluation for possible immune dysfunction. Subsequent clinical, radiological, and microbiological findings supported a clinical diagnosis of PJP requiring treatment. It conveys three clinically important messages.

First, postoperative CRS accompanied by persistent adult otitis media should not be solely attributed to residual local disease; rather, an underlying systemic disorder should be promptly considered. In the present case, the patient continued to experience purulent rhinorrhea and postnasal drip despite undergoing ESS twice and subsequently developed prolonged otorrhea. Such a course is atypical in ordinary CRS management, and clinicians should reconsider the diagnostic framework more broadly in this setting.

Second, *Pseudomonas aeruginosa* was detected. *Pseudomonas aeruginosa* is a recognized organism in postoperative or recurrent rhinosinusitis and otitis media [6]. In this patient, *Pseudomonas aeruginosa* was isolated from both nasal discharge and otorrhea. Rather than indicating immune dysfunction by itself, the microbiological finding was considered together with persistent infection despite two sinus surgeries, adult-onset otitis media, and a history of hematologic malignancy and rituximab exposure. This overall clinical constellation warranted consideration of an underlying immune disorder [7].

Third, the sinonasal and otologic symptoms preceded clinical recognition of the pulmonary infection. During hospitalization, evaluation of hypoxemia, chest CT abnormalities, elevated β-D-glucan, and sputum PCR findings supported the clinical diagnosis of PJP. Persistent CRS and otitis media or persistent upper-airway infection may thus provide an opportunity for earlier immunologic and pulmonary diagnostic evaluation.

The underlying immune disorder should be interpreted cautiously. The patient had hypogammaglobulinemia and reduced CD4/CD8 counts, but the etiology remained undetermined. Although he had received three cycles of R-CHOP approximately 10 years earlier, the interval since treatment neither excludes prolonged rituximab-associated secondary hypogammaglobulinemia nor establishes a primary immunodeficiency. Common variable immunodeficiency was considered in the differential diagnosis, but a definitive diagnosis was not justified without a complete evaluation of secondary causes, functional antibody responses, and B-cell subsets [8]. Furthermore, CD4/CD8 subsets were measured after systemic corticosteroids had been initiated. Thus, corticosteroid-associated lymphopenia and acute illness may have influenced these findings, and the pretreatment cellular immune status cannot be established. This case has several limitations. PJP was not microbiologically confirmed by bronchoalveolar lavage or microscopic demonstration of the organism. Neither room-air arterial blood gas values nor pretreatment CD4/CD8 counts were available. Molecular typing was not performed on the nasal and otologic *P. aeruginosa* isolates, and the primary versus secondary etiology of hypogammaglobulinemia remained undetermined. Although six-week immunoglobulin data were available, comprehensive follow-up after transfer to another hospital was not available.

## 4 Conclusion

In patients with persistent or treatment-refractory postoperative CRS accompanied by adult-onset otitis media, clinicians should consider underlying immune dysfunction, particularly when there is a history of hematologic malignancy or immunomodulatory therapy. The combined clinical features may prompt earlier immunologic and pulmonary diagnostic evaluation for opportunistic infection.

## CRediT authorship contribution statement

**Ryota Higuchi:** Writing – original draft, Investigation, Formal analysis, Data curation, Conceptualization. **Yusuke Miyamoto:** Supervision. **Kenichi Nakao:** Writing – review & editing, Validation. **Shunsuke Tanaka:** Investigation, Data curation. **Yoshihiro Eriguchi:** Writing – review & editing, Supervision. **Takashi Nakagawa:** Supervision.

## Informed consent statement

Written informed consent was obtained from the patient for publication of this case report and the accompanying images.

## Institutional Ethics/IRB approval statement

This case report was approved by the Institutional Review Board of our institution under Clinical Research No. 2021-346.

## Declaration of generative AI and AI-assisted technologies in the manuscript preparation process

During the preparation of this work, the author(s) used ChatGPT to perform the English translation. The author(s) reviewed and edited the output as needed and take full responsibility for the content of the published article.

## Funding statement

This work was supported by a research grant from The Soda Toyoji 10.13039/100028926Memorial Foundation.

## Declaration of competing interest

The authors have no conflicts of interest to declare.
