# Supplementary material for: Refractory postoperative chronic rhinosinusitis and otitis media as the initial clue to hypogammaglobulinemia and Pneumocystis jirovecii pneumonia: A case report
Source: Respir Med Case Rep. 2026 Oct 27;64:102536. doi: 10.1016/j.rmcr.2026.102536 (PMC13742234; doi:10.1016/j.rmcr.2026.102536)
Supplement: Supplementary file 1 [file mmc1.pdf]

CARE case report guidelines

CARE Checklist of information to include when writing a case report

(cc) BY-NC-ND

Topic	Item	Checklist item description	Reported on Line
Title	1	The diagnosis or intervention of primary focus followed by the words "case report"	Page 1, Lines 3-4
Key Words	2	2 to 5 key words that identify diagnoses or interventions in this case report, including "case report"	Page 2, Lines 21-22
Abstract (no references)	3a	Introduction: What is unique about this case and what does it add to the scientific literature?	Page 2, Lines 2-4, 18-19
	3b	Main symptoms and/or important clinical findings	Page 2, Lines 4-9
	3c	The main diagnoses, therapeutic interventions, and outcomes	Page 2, Lines 8-17
	3d	Conclusion—What is the main "take-away" lesson(s) from this case?	Page 2, Lines 17-19
Introduction	4	One or two paragraphs summarizing why this case is unique (**may include references**)	Page 3, Lines 3-13
Patient Information	5a	De-identified patient specific information.	Page 3, Lines 16-21
	5b	Primary concerns and symptoms of the patient.	Page 3, Lines 16-17
	5c	Medical, family, and psycho-social history including relevant genetic information	Page 3, Lines 17-21
	5d	Relevant past interventions with outcomes	Page 3, Lines 18-25
Clinical Findings	6	Describe significant physical examination (PE) and important clinical findings.	Page 4, Lines 1-6, 12-18
Timeline	7	Historical and current information from this episode of care organized as a timeline	Page 10, Lines 16-19; Table 2
Diagnostic Assessment	8a	Diagnostic testing (such as PE, laboratory testing, imaging, surveys).	Page 4, Lines 1-6, 14-26
	8b	Diagnostic challenges (such as access to testing, financial, or cultural)	Page 6, Lines 21-26
	8c	Diagnosis (including other diagnoses considered)	Page 4, L23-26; Page 6, L14-20
	8d	Prognosis (such as staging in oncology) where applicable	N/A
Therapeutic Intervention	9a	Types of therapeutic intervention (such as pharmacologic, surgical, preventive, self-care)	Page 4, L7-11; Page 5, L4-12
	9b	Administration of therapeutic intervention (such as dosage, strength, duration)	Page 4, L10-11; Page 5, L4-12
	9c	Changes in therapeutic intervention (with rationale)	Page 5, Lines 4-8
Follow-up and Outcomes	10a	Clinician and patient-assessed outcomes (if available)	Page 5, Lines 8-15
	10b	Important follow-up diagnostic and other test results	Page 4, L19-21; Page 5, L11-15
	10c	Intervention adherence and tolerability (How was this assessed?)	Page 5, Lines 4-8
	10d	Adverse and unanticipated events	Page 5, Lines 5-7
Discussion	11a	A scientific discussion of the strengths AND limitations associated with this case report	Page 6, Lines 14-26
	11b	Discussion of the relevant medical literature **with references**.	Page 6, Lines 3-8, 14-20
	11c	The scientific rationale for any conclusions (including assessment of possible causes)	Page 6, Lines 14-23
	11d	The primary "take-away" lessons of this case report (without references) in a one paragraph conclusion	Page 7, Lines 4-8
Patient Perspective	12	The patient should share their perspective in one to two paragraphs on the treatment(s) they received	Not reported
Informed Consent	13	Did the patient give informed consent? Please provide if requested	Yes ☑ No ☐

Item 13 location: Page 8, Lines 2-4
